# Mid-infrared acetone gas sensors using substrate-integrated hollow waveguides augmented by advanced preconcentrators

**DOI:** 10.1038/s41598-025-02514-w

**Published:** 2025-05-29

**Authors:** Diandra Nunes Barreto, Carmen Schreiber, João Flávio da Silveira Petruci, Boris Mizaikoff, Vjekoslav Kokoric

**Affiliations:** 1 Institute of Analytical and Bioanalytical Chemistry, Ulm University, 89081 Ulm, Germany; 2 Hahn-Schickard, 89077 Ulm, Germany; 3 Institute of Chemistry, Federal University of Uberlândia (UFU), Uberlândia, MG Brazil

**Keywords:** MIR, Breath analysis, Direct analysis, Preconcentration, VOCs, Infrared spectroscopy, Sensors

## Abstract

The identification of volatile organic compounds (VOC) such as acetone, a relevant biomarker for diabetes mellitus in exhaled human breath has become essential for early disease diagnostics, prognosis, and monitoring of metabolic responses to pharmacological interventions. Gas chromatography coupled with mass spectrometry (GC–MS) is considered as the gold standard for breath analysis. However, its inability to offer point-of-care monitoring limits its applicability in clinical environments. Optical techniques based on absorption in the mid-infrared range (2.5 to 25 μm) appear as promising alternatives due to their inherent selectivity, potential for miniaturization, portability, and direct analysis with rapid response. Relevant biomarkers present in exhaled breath, are usually observed in the ppb to ppm (v/v) concentration regime. For sensitivity enhancement of optical sensing techniques, appropriate preconcentration schemes are required prior to the optical detection. The present study describes a method combining a multi-channel substrate-integrated preconcentration module (a.k.a., muciPRECON) for enhancing and optimizing the detection of acetone via a mid-infrared photonic sensing system. The sensor system comprises a compact Fourier Transform Infrared (FTIR) spectrometer, a technique that enables detailed infrared spectral analysis, coupled to a substrate-integrated hollow waveguide (iHWG) simultaneously acting as a gas cell and as a photon conduit. Preconcentation experiments were from acetone/nitrogen gas mixtures in the concentration range of 5–100 ppm at − 10 °C followed by desorption at a temperature of 100 °C and direct injection into the IR sensing system Thus obtained acetone spectra were quantified evaluating a molecule-specific vibrational absorption peak area in the spectral window 1260–1170 cm^−1^. After extensive screening, Tenax was identified as superior sorbent material providing an enrichment factor of up to 153-times, a limit of detection (LOD) of 0.118 ppm, and a limit of quantification (LOQ) of 0.393 ppm. These results are indeed promising for practical applications, especially since acetone concentrations usually vary between 0.3 and 0.9 ppmv within the exhaled breath of healthy individuals, and in individuals with diabetes, acetone concentrations are typically around 1.7 to 3.0 ppmv. Consequently, the developed systems have the necessary sensitivity and accuracy to detect acetone levels that are in the relevant physiological range indicating their potential use in future real-world scenarios.

## Introduction

Breath analysis provides a non-invasive and effective method of detecting a large number of chemical species in exhaled breath (EB). More than 500 compounds have been identified offering valuable information on physiological conditions and disease states via direct detection and real-time monitoring. This entails a significant potential in medical diagnosis^1,2^. Among the volatile organic compounds (VOCs) found in the EB, acetone is a relevant biomarker related to various conditions including but not limited to diabetes, ketogenic diets, liver disease and metabolic disorders. The accurate quantification of acetone is crucial for early disease detection and the implementation of effective treatment strategies for patients^3–5^.

Generally, the gold-standard analytical techniques for breath analysis are gas chromatography coupled with mass spectrometry (GC-MS) and ion mobility spectrometry (IMS)^3^. Additionally, mass spectrometry has advanced significantly, particularly with the development of ambient ionization techniques. In disease diagnostics, especially in the analysis of volatile compounds in breath, ambient ionization techniques facilitate rapid and highly sensitive biomarker detection^6^. However, a major drawback of these techniques is the potential for background interferences, which can complicate result interpretation, as direct ionization may also detect non-target compounds in the complex sample matrix, reducing detection specificity.

Alternatively, optical-based techniques such as cavity ringdown spectroscopy (CRDS) photoacoustic spectroscopy (PAS), and laser absorption spectroscopy (LAS) have been utilized to quantify acetone in breath samples^2^. These techniques offer high sensitivity, selectivity, and accuracy, making them efficient tools for gas detection across various applications, including medical/clinical analysis and environmental monitoring^13,14^. Nevertheless, the bulky dimensions and high costs of these instruments make them less feasible for point-of-care (POC) or point-of-need (PON) settings, and they do not support online, real-time monitoring^1,8^. Furthermore, these methods have limitations that may affect their practicality. For instance, issues with cavity alignment in cavity-enhanced techniques can compromise measurement accuracy and reliability^7^. Additionally, a limitation of PAS is potential interference from broadband absorption by molecules other than the target analyte, which can lead to elevated background signals and reduced sensitivity^8^. Thus, alternative optical sensing strategies are necessary to enhance the portability and accessibility of these techniques, making them more practical for real-world applications, such as air quality monitoring and the early diagnosis of diseases via breath analysis.

Mid-infrared (MIR) photonics stand out for their ability to identify molecules/ target analytes based on their unique absorption spectra in the MIR region. This specificity results from distinct vibrational and rotational transitions of molecules in the MIR range providing a unique signature for each molecule and allowing precise discrimination of different gases even in complex samples^9^.

Furthermore, MIR spectroscopy provides molecular fingerprints enabling the identification of various compounds based on their unique spectral characteristics. In addition, MIR spectroscopy enables portable sensors for analyzing gas samples online and in real-time ideally suited for POC/PON exhaled air measurements^10^.

The implementation of miniaturized gas cells, i.e., so-called substrate-integrated hollow waveguides (iHWG) pioneered by the Mizaikoff research team provides an adaptable (i.e., designable) gas cell concept that allows the gaseous sample to reproducibly interact with radiation while guiding the incident light to the detector. Hence, the analysis of gaseous and volatile samples is enabled close to real-time^11,12^. The modular concept of the iHWG contributes to the miniaturized footprint of the optical sensor system when coupled to portable spectrometers or light sources across the analytically useful electromagnetic window from the MIR to the UV^13^.

However, most volatile biomarkers in EB are present at low concentrations, typically in the ppm-to-ppb range. For this reason, a preconcentration step is frequently required to enrich the sample prior to analysis^14^. Adsorption onto solid sorbent materials is considered the most efficient, solvent-free method for capturing volatile constituents. Preconcentration techniques effectively enhance detection sensitivity, especially in systems based on optical absorbance spectroscopies^15^. The trapping efficiency, however, is significantly influenced by the choice of solid sorbent. Therefore, accurately detecting trace levels within reasonably short sampling times requires selecting ideal sorbents for the specific target analytes—such as when preconcentrating acetone and other relevant molecules^16–18^.

Kokoric et al. have developed an innovative preconcentration system, the so-called muciPRECON, which offers three individual sorbent channels integrated into an aluminum substrate. The muciPRECON system offers enhanced capabilities for the simultaneous preconcentration of a variety of VOCs present in complex gas sample^16^. This versatile and efficient method allows for the sensitive and accurate detection of analytes that is readily adaptable to a wide variety of application scenarios with portable dimensions, and a selectable number of channels and sorbents that can be coupled to useful detection techniques^16^.

The present study investigated acetone enrichment via various sorbent materials using a multi-channel preconcentrator to optimize the detectivity of this target species via mid-infrared photonics. The muciPRECON was connected to an MIR photonic sensing system based on an FTIR spectrometer coupled to a substrate-integrated hollow waveguide and an MCT detector. After recording absorbance spectra, the peak area between 1260 and 1170 cm^−1^ was evaluated for quantification purposes.

## Methods

### Photonic sensor system

**Fig. 1:**
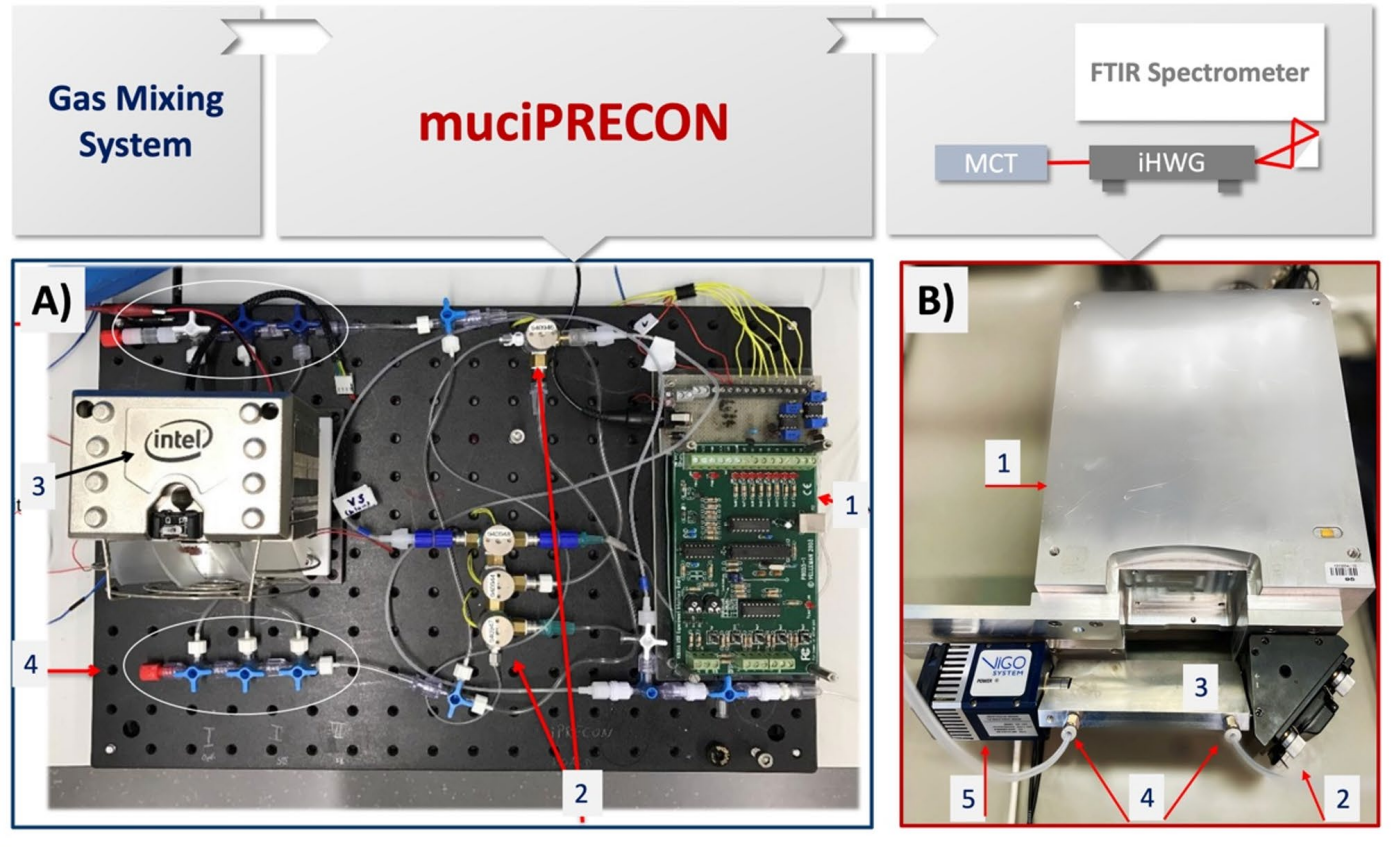
*Top*: Schematic of the experimental setup. *Bottom*: Photographs of the main components. (**A**) The muciPRECON module with the following components: (A.1) valve control interface; (A.2) magnetic valves; (A.3) CPU cooler for thermal regulation; (A.4) manual valves for selecting the sorbent channels. (**B**) The detector system: (B.1) infrared source (FTIR spectrometer); (B.2) gold-coated off-axis parabolic mirror (OAPM); (B.3) straight-line iHWG; (B.4) gas inlet/outlet; (B.5) MCT detector for signal acquisition.

A schematic of the experimental setup (muciPRECON and MIR sensing device) is shown in Fig. 1. All measurements were performed using a portable FTIR spectrometer (Alpha OEM, Bruker Optics Inc., Ettlingen, Germany) as light source (B.1). The IR radiation was focused using a gold coated off-axis parabolic mirror (B.2) with a focal length of 1 inch. (Thorlabs, Dachau, Germany) into the iHWG (B.3) made from polished aluminium (with dimensions of 150 × 25 × 15 mm (length × width × depth) with entrance ports sealed with BaF_2_ IR-transparent windows, (B.4) cooled MCT detector (PVM-4TE-8, Vigo SA, Poland) with (FT-IR-22.1.00, Infrared Associates, Stuart, FL) was positioned at the distal end of the iHWG to detect the signal after photon-molecule interaction. Gas standards for calibrations were prepared using a gas mixing system (GMS; developed in collaboration with Lawrence Livermore National Laboratory, Livermore, CA, USA). An acetone standard at a concentration of 100 ppmv was utilized to prepare a series of diluted solutions at various concentrations using nitrogen (obtained from MTI Industriegase AG, Neu-Ulm, Germany). (A) The muciPRECON module was positioned between the gas mixing system and the photonic detection system containing different polymeric adsorbents including Optipore V493, Tenax TA, HayesepD, and Porapak QS (obtained from Sigma-Aldrich, St. Louis, MO).

The schematic in Fig. 1 shows the direction of the gas flow initiated by the GMS, i.e., the carrier gas (N_2_) path and the acetone measurement cycle. The spectra were recorded in the wavelength range of 4000–400 cm^−1^ at a spectral resolution of 2 cm^−1^. The OPUS 7.2 software package (Bruker Optics Inc., Ettlingen, Germany) was used for data acquisition.

### Multi-channel preconcentrator (muciPRECON)

**Fig. 2:**
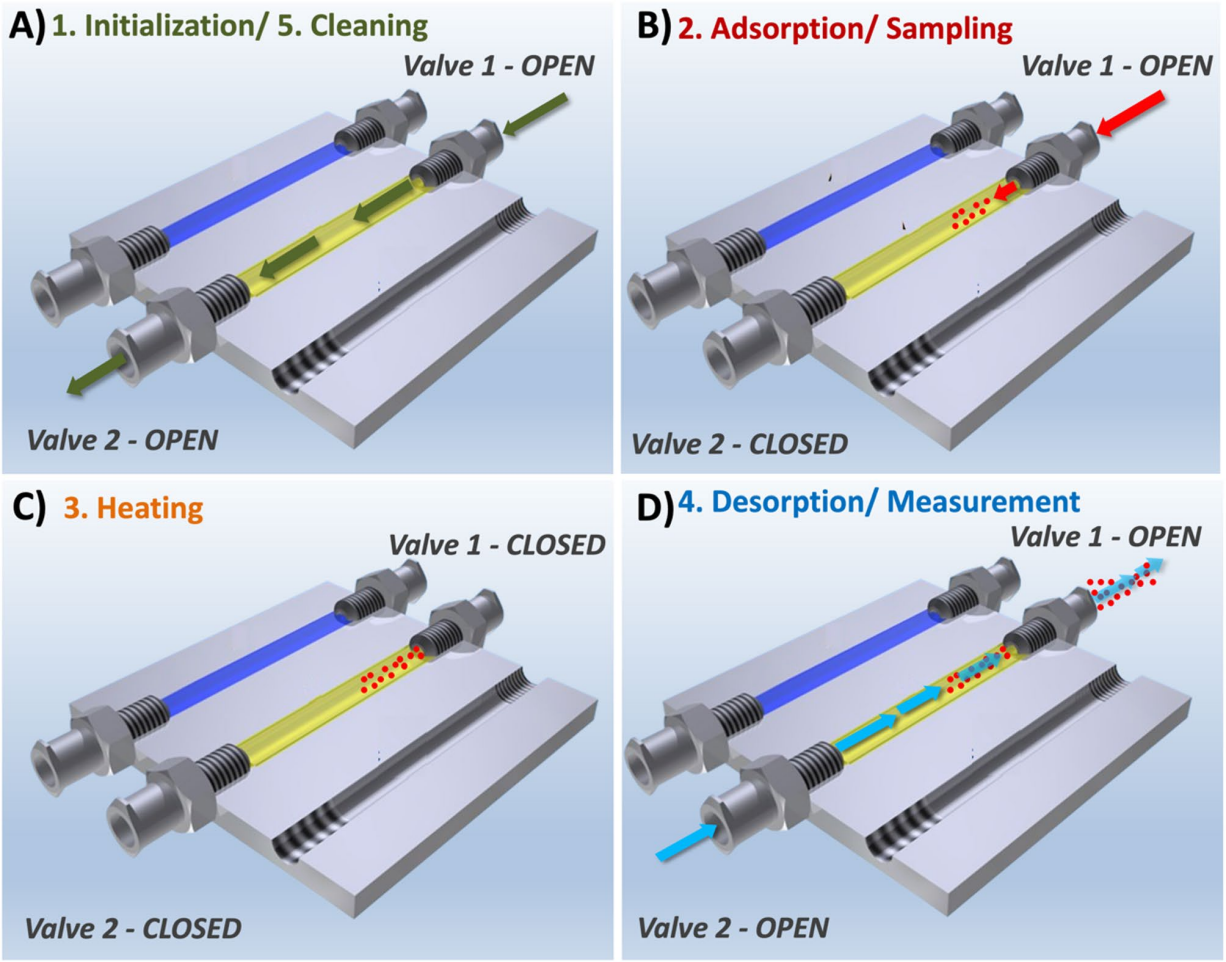
Illustration of gas flow and directions at each operational stage: (**A**) Start-up/Cleaning: The green arrow indicates the gas flow during the initial set-up and cleaning phase, with Valve 1 and 2 open. (**B**) Adsorption/Sampling: The red arrow represents the gas flow through the adsorbent material for sampling, with Valve 1 open and Valve 2 closed. (**C**) Heating: During this phase, the system is isolated when both valves are closed and the adsorbent is heated to prepare for desorption. (**D**) Desorption/Measurement: The blue arrow shows the gas flow in the measurement phase, in which the desorbed molecules are directed to the detection system, with valves 1 and 2 open.

The muciPRECON device (Fig. 2) has three channels packed with different adsorbent materials for the targeted extraction of VOCs from complex vapor phase samples such as EB. These three channels can be connected sequentially allowing for the vapor phase sample to pass through each channel or may also be operated individually / in parallel to divide the sample flow into three equal parts.

The adsorption and desorption steps are carried out at different temperatures and in different flow directions. Peltier elements were incorporated facilitating thermoelectric heating and cooling for temperature control enabling sampling, cleaning, and desorption. This device is possibly compact (L x W x D: 5 × 5 × 0.7 cm) and allows temperature control and manipulating the flow direction at each stage fully automated in a compact platform.

### Analysis procedure

The procedure involves five distinct stages, all controlled by the muciPRECON control software (developed in-house in Visual Basic; Microsoft., Albuquerque, NM, US). The software manages the magnetic valves (NResearch Inc., West Caldwell, NJ, USA), allowing open and close in the correct directions. The software also regulates the temperatures at all the stages involved in the analysis processes. The description of each stage is detailed below and illustrated in Fig. 2.

#### Initialization

First, the muciPRECON is heated to 100 °C and purged with nitrogen at a flow rate of 200 mL min^−1^ through each channel for 10 min. This step is needed to remove residual acetone and other volatile compounds that may remain adsorbed on the sorbent material from previous cycles or from manufacturing. Heating up to 100 °C promotes the desorption of these species without compromising the physical integrity of the sorbents, as this temperature is within the recommended operating range for materials such as Tenax TA. In addition, thermal activation at this stage ensures that the adsorption sites are available for interaction with new acetone molecules, contributing to consistent adsorption performance in subsequent analyses.

#### Sampling cycle

In this step, acetone is adsorbed onto the absorbent material and muciPRECON is cooled to − 10 °C before starting the enrichment process. Reducing the temperature improves the interaction between acetone molecules and the adsorbent, reducing the volatility of the analyte and increasing its residence time in the porous matrix. The gas mixture containing acetone flows through the channel in the reverse direction to the initialization step, at a flow rate of 200 mL min^−1^ for 10 min. After the sampling period, all the valves are closed manually and the desorption cycle is started, as indicated by the red arrow in Fig. 2B.

#### Heating cycle

At this stage, the software initiates the “Measurement” process and activates the heater. Once the muciPRECON reaches 90 °C, the software directs a nitrogen flow of 10 mL min^−1^ directly into the iHWG omitting the muciPRECON to clean the optical path and for recording a background spectrum. Meanwhile, the enriched acetone is desorbed due to the applied heat. The valves remain closed without flow to the muciPRECON. Only background measurements with nitrogen commence.

#### Spectral data acquisition

The flow rate is maintained at 10 mL min^−1^ and the initial acetone measurement initiates once the desired temperature of 100 °C is achieved. In the next step, the valves are opened and the nitrogen gas flow passes though the adsorption channel of the muciPRECON. Desorbed acetone molecules are transported to the MIR setup. It is worth noting that the flow through the channel is in opposite direction vs. the sampling process based on the principle that enrichment occurs primarily at the beginning of the channel. These settings are maintained until the measurement is completed. Thus, for each acetone preconcentration cycle, 40 repeated measurements are recorded with 8 averaged scans at a spectral resolution of 2 cm^−1^.

#### Cleaning/purging (muciPRECON and iHWG).

After the measurements have been completed, the final stage consists of cleaning the system. The temperature is maintained at constant 100 °C, and each channel used is purged with nitrogen for 5 min at a flow rate of 100 mL min^−1^.

**Fig. 3:**
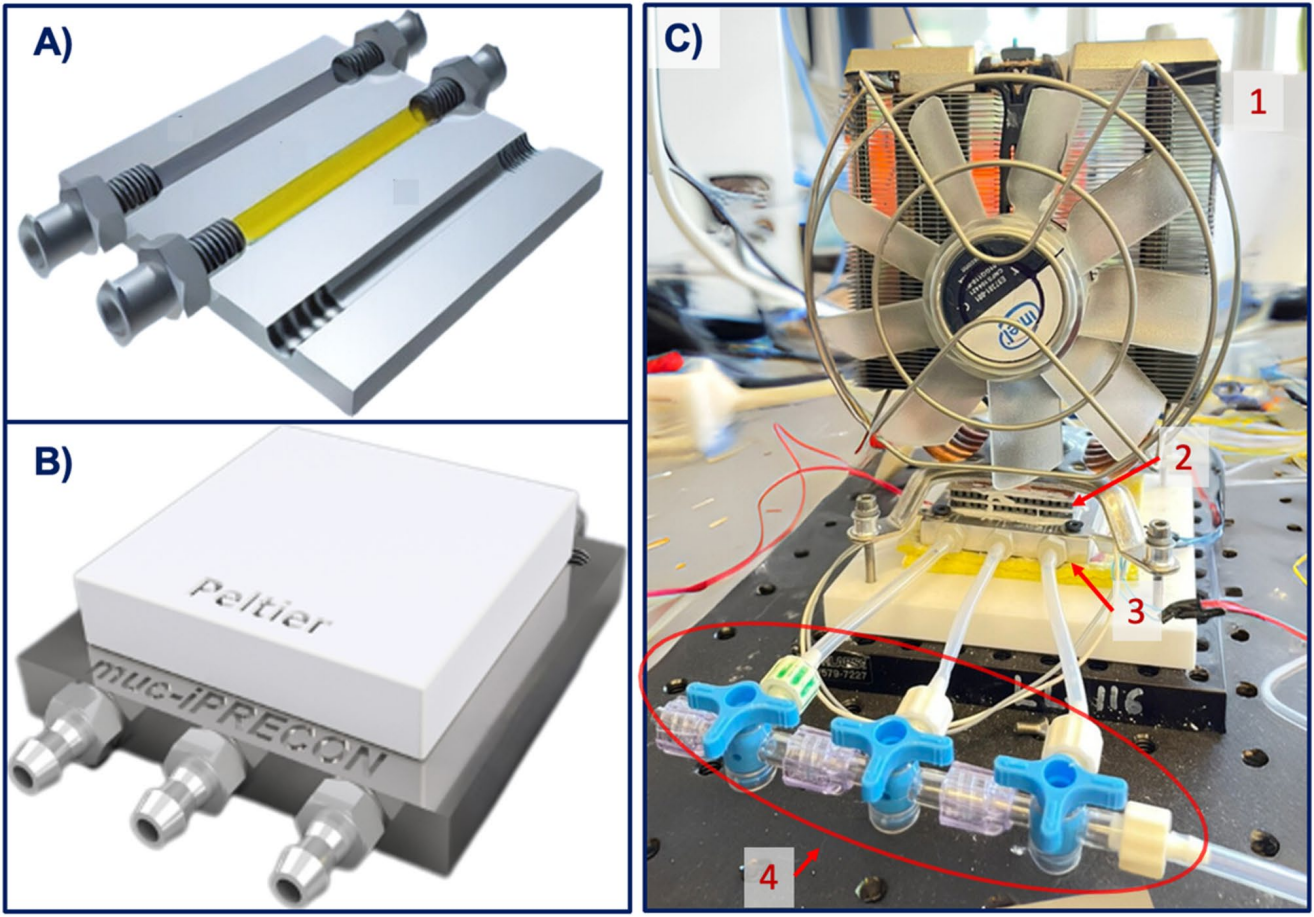
(**A**) 3D rendering of the muciPRECON, illustrating its internal channels connected to Luer locks; (**B**) 3D view of the closed muciPRECON with a Peltier element mounted on top for temperature control; (**C**) photograph of the assembled prototype. In (C.1) CPU cooler for overall temperature management; (C.2) metal substrate containing the muciPRECON channels; (C.3) Peltier element to control the temperature of each stage; (C.4) manual valves for selecting the sorbent material.

The structure and operation of the muciPRECON system are illustrated in Fig. 3(A–C). Figure 3A is a 3D schematic model of the device’s internal channels, which are 1 mm in diameter and housed in a metal block. Figure 3B shows the assembled module with a Peltier element mounted on the top surface, which is responsible for cooling the sorbent region to − 10 °C during the adsorption phase. Figure 3C shows a photograph of the complete configuration, in which: (C.1) a CPU fan and heat sink are used to dissipate heat from the hot side of the Peltier and stabilize the system temperature; (C.2) the metal base contains the embedded sorbent channels; (C.3) the Peltier device provides localized thermal control for cooling and subsequent heating; and (C.4) a set of three-way manual valves is used to select the active channel and isolate the others. During the start-up and desorption phases, the selected channel is connected in direct flow mode, while during the sampling cycle, the flow direction is reversed and the other valves remain closed to ensure that only one absorbent material is used per cycle. This configuration allows precise thermal and flow management for effective preconcentration and desorption of volatile compounds.

## Results

### Infrared spectrum of acetone

**Fig. 4:**
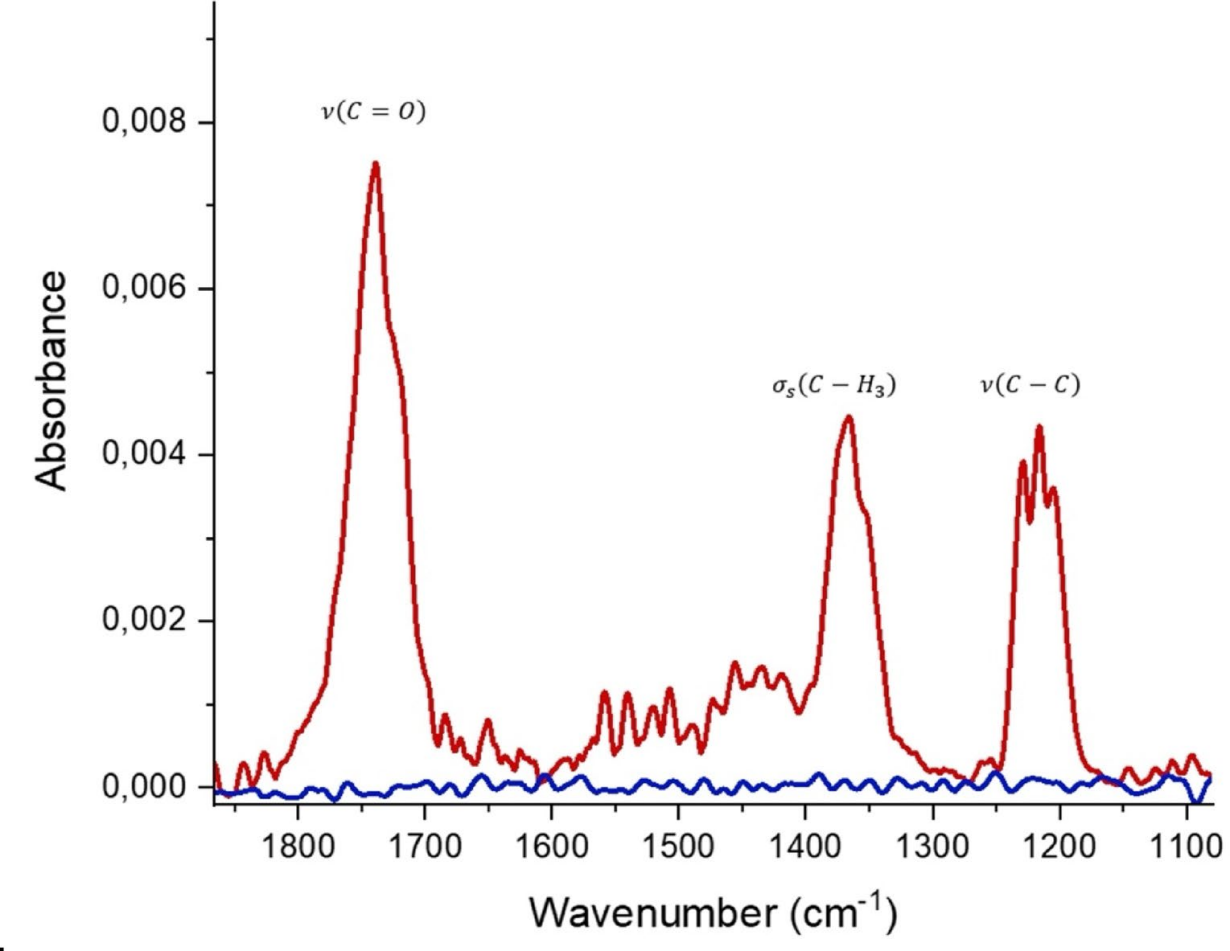
(red) Absorption spectrum of 100 ppmv acetone; (blue) Background spectrum with N_2_ present within the iHWG channel. Both spectra result from 8 averaged scans at a spectral resolution of 2 cm^−1^.

The spectral signature of acetone in the MIR region includes several distinct absorption bands related to different molecular vibrations. Figure 4 shows the IR spectrum of a 100 ppm acetone sample without preconcentration. Deformation vibrations of the methyl groups appear at 1350–1470 cm^−1^ with specific peaks at 1375 cm^−1^ for the symmetric deformation and 1450 cm^−1^ for the asymmetric deformation. The 1725 cm^−1^ band corresponding to the carbonyl (C=O) stretching vibration is particularly prominent due to its intensity resulting from the polarity of the carbonyl group. However, this region may show spectral interference from aldehydes (1725–1740 cm^−1^), water vapor, and CO_2_. The C–C skeletal stretching vibrations occur between 900 and 1200 cm^−1^ but are weaker and less specific. Therefore, the absorption band between 1260 and 1170 cm^−1^ was used to evaluate the data because it is less affected by interfering species, thus enabling accurate detection of acetone in complex matrices, such as exhaled air^19–21^.

### Sorbent materials

**Fig. 5:**
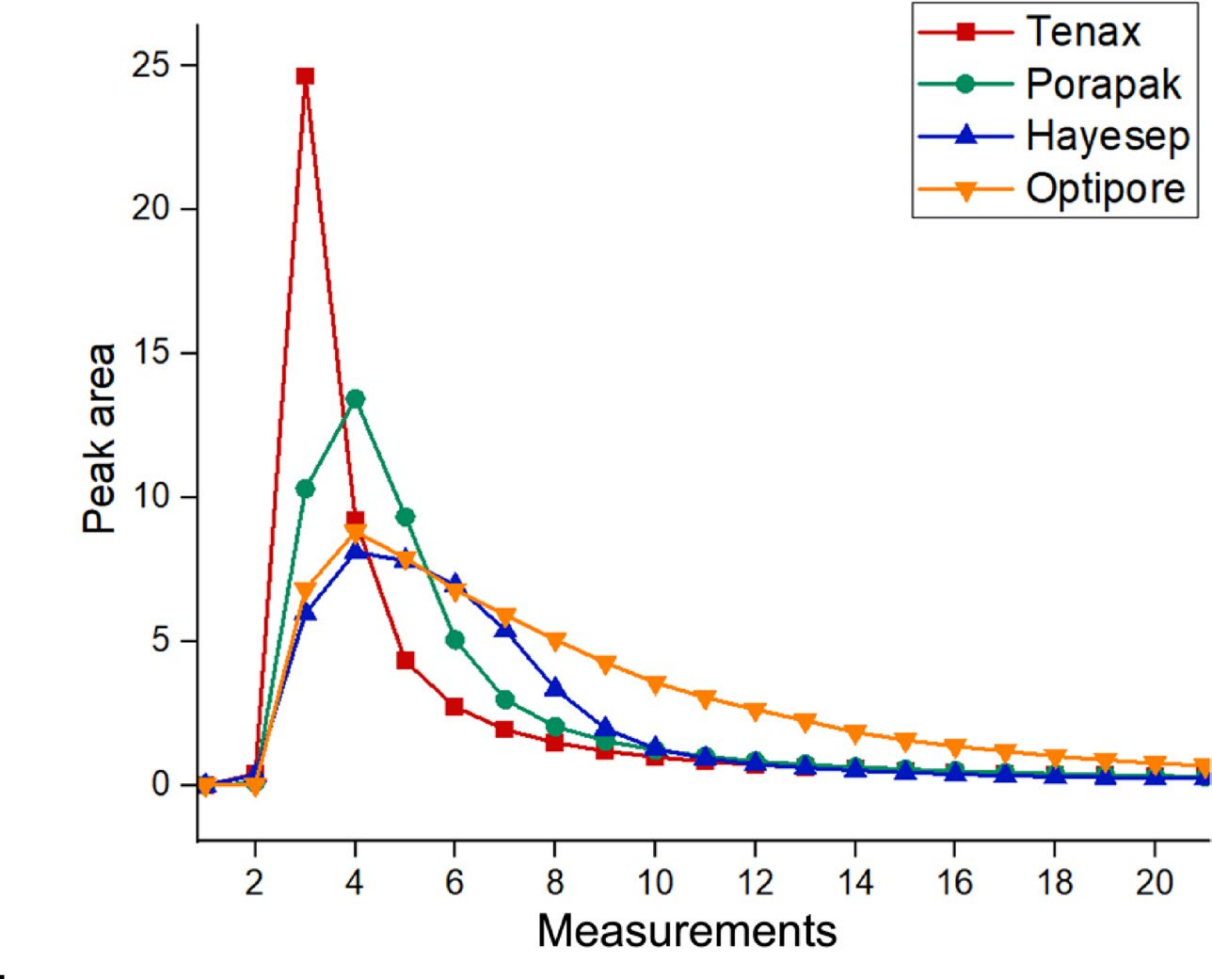
Desorption profiles versus number of spectrum for each sorbent material obtained via the MIR sensor system evaluating the peak area in the spectral range 1260–1170 cm^−1^.

The surface characteristics of the sorbent materials were evaluated, focusing on key parameters such as porosity, particle diameter, surface area, and uniformity, all of which are crucial for the effectiveness of the sorption process^22^. The specific surface area (m^2^ g^−1^) was obtained from the technical datasheets provided by the manufacturers, and the theoretical surface area (m²) per channel was calculated by multiplying this value by the mass used^23^. These values are summarized in Table 1. The absorbent materials were used in different masses due to the physical constraints of the muciPRECON channels, which led to variability in the total surface area available between the materials. To allow for a more balanced comparison, the adsorption performance was evaluated by calculating the response per gram of adsorbent (area/g), based on the peak area observed in the desorption profile (Fig. 5) divided by the respective mass of material used. This normalization provides a more consistent interpretation of the adsorption capacity in relation to the amount of adsorbent applied.

Among the evaluated sorbent materials, Optipore, with its hypercrosslinked polystyrene-based structure, forms a network of micropores with a high surface area, making it ideal for adsorbing larger, less volatile organic compounds^24^. However, its lack of specific functional groups limits its interactions with small, polar molecules In this context, Porapak QS, a highly crosslinked polystyrene sorbent, features a structure that offers a large surface area and an efficient pore network for capturing volatile organic compounds (VOCs)^22^. Its porous structure allows for effective mass transfer and high adsorption capacity, making it especially suitable for the preconcentration of VOCs from both gaseous and liquid matrices. This material is widely used in environmental monitoring and chemical analysis, efficiently adsorbing VOCs due to favorable interactions and excellent analyte retention^25^.

In another hand, Tenax is a polymeric sorbent primarily composed of poly(2,6-diphenyl-p-phenylene oxide), is characterized by a highly crosslinked, rigid matrix that provides thermal stability and hydrophobic properties. This structure allows Tenax to effectively adsorb VOCs, such as hydrocarbons and ketones, particularly in humid environments, due to its low affinity for water. Its stability and efficient adsorption make Tenax a popular choice for air and breath sample analyses, excelling in both environmental monitoring and chemical analysis applications^26,27^.

Lastly, Hayesep D, a macroporous divinylbenzene resin, is known for its high surface area and large pore volume, providing excellent adsorption capabilities for light gases. Its structure facilitates efficient mass transfer and rapid adsorption, making it ideal for dynamic sampling applications. Hayesep D is particularly effective in capturing gases like methane and nitrous oxide, and it can selectively adsorb trace gases, such as halogenated greenhouse gases, while minimizing interference from other substances. Its versatile performance in environmental monitoring and gas chromatography underscores its significant role in analytical chemistry^28^.

**Table 1:** Mass of sorbent material packed into the preconcentration channels of the MuciPRECON along with their respective total and theoretical surface area.

Sorbent	Mass used in muciPRECON (g)	Surface m^2^ g^−1^	Theoretical surface m^2^	Adsorption efficiency area g^−1^
Optipore V493	0.1580	1100	173.80	14.68
Porapak QS	0.1440	550	79.20	12.84
Tenax	0.0838	35	2.93	22.66
Hayesep D	0.1598	795	126.64	9.99

The adsorption efficiency normalized by mass (area-g^−1^) compares the adsorbents regardless of differences in mass or surface area. Tenax showed the highest normalized efficiency, despite having the lowest theoretical surface area, indicating good performance in relation to the amount used. Therefore, to compare the adsorbent materials, the desorption profiles recorded by the MIR sensor system were analyzed individually. The data points represent the average of 10 measurements obtained by analyzing the peak area in the 1260–1170 cm^−1^ region. The enrichment step was conducted under consistent conditions: 100 ppm acetone at a flow rate of 200 ml/min for 10 min during the enrichment step. To compare the adsorbent materials and evaluate the desorption dynamics, IR spectra were sequentially acquired using 8 scans per spectrum, where each scan corresponds to approximately 2 s and, therefore, each spectrum is recorded after 16 s. Figure 5 presents the peak area obtained in each spectrum and it can be seen that the most significant signal occurred between the second and sixth measurements. After the acquisition of 18 spectra, no further acetone absorbance signals were detected, suggesting that complete desorption had occurred for all adsorbents.

Among the evaluated materials, Tenax exhibited the highest peak area after the third measurment, indicating a rapid and intense desorption, followed by Porapak, Hayasep, and Optipore. The differences in desorption profiles reflect the varying capacities and affinities of each sorbent material for acetone. These results were used to select the optimal scan range for subsequent analyses.

Surface area, pore size distribution, and porosity are critical parameters that influence the adsorption capacity and efficiency of an adsorbent material. A larger surface area provides more active sites for adsorption, leading to an increased adsorption capacity. The pore size distribution is particularly important as it dictates the types and sizes of molecules that can be effectively adsorbed. Additionally, an optimized adsorption process should feature high selectivity, fast desorption kinetics, and complete desorption of the target molecules from the adsorbent. For VOC preconcentration applications, an ideal adsorbent should also facilitate desorption at moderate temperatures, as evidenced by the Tenax sorbent. After three scan cycles, a noticeable decrease in the signal is observed, indicating rapid desorption of the material. In addition, the lack of signal is also observed in scans 1 and 2, which is due to the automated system, which starts detection as quickly as the muciPRECON measurement process begins. However, the signal is only detected after the second scan because it takes time for the sample to make the transition from the pre-concentration system to the IR detection system. The analyte only reaches the detection system after the first two scans, allowing the signal to be recorded.

In other hands, adsorbents with a large surface area such as Optipore provide higher adsorption capacities due to the increased interaction area between the material and the molecules. However, during the desorption process higher temperatures are usually required to completely desorb the adsorbed molecules allowing analysis and subsequent reuse of the adsorbent.

## Discussion

**Fig. 6:**
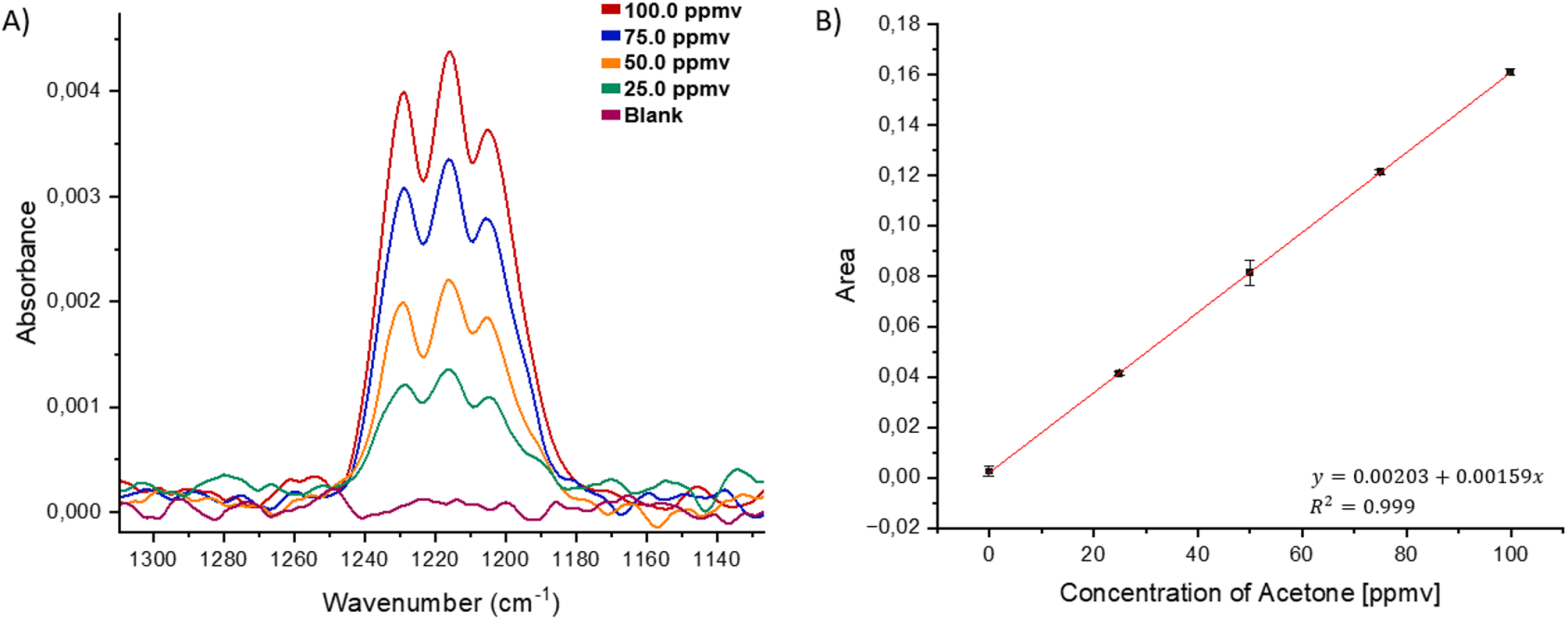
(**A**) Acetone spectra at different concentrations using the MIR sensing device, averaging 8 scans at a spectral resolution of 2 cm^−1^, without preconcentration. (**B**) Acetone calibration curve without preconcentration (R^2^ = 0.999). The calibration curve was constructed using three measurements for each concentration point.

The efficiency of acetone preconcentration using these absorbent materials was evaluated establishing calibration functions with preconcentration vs. calibrations obtained without preconcentration (Fig. 6).

**Fig. 7:**
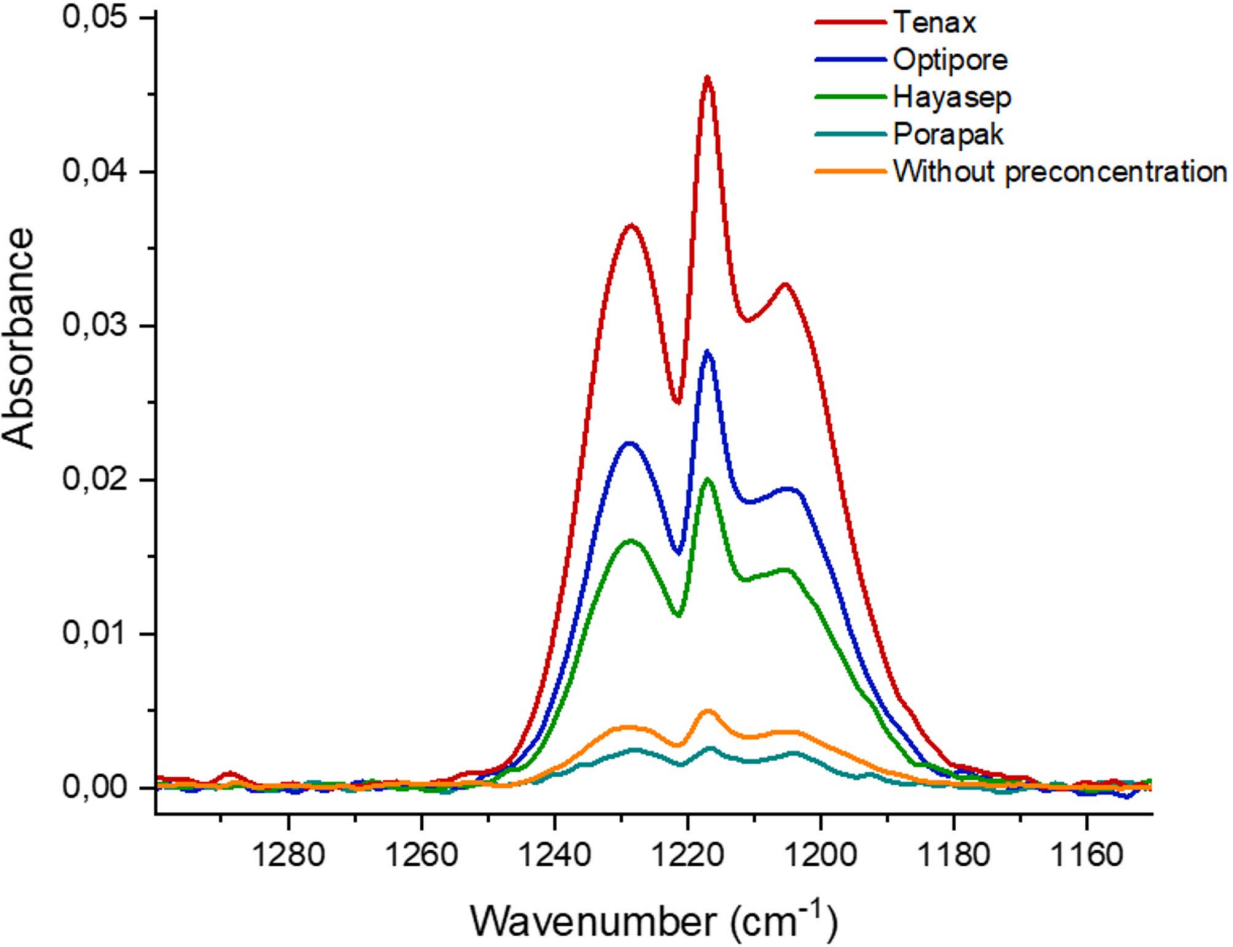
IR spectrum of 100 ppmv acetone without preconcentration compared to different sorbent materials.

The concentration of acetone in the breath of healthy individuals has been reported to range from 0.3 to 0.9 ppmv, while in individuals with diabetes, acetone concentrations are typically around 1.7 to 3.0 ppmv^2,29^. However, MIR sensors that operate without pre-concentration strategies reach limits of detection (LOD) and quantification (LOQ) of 15.9 ppmv and 59.07 ppmv, respectively, which are inadequate for accurately detecting the relevant acetone levels in exhaled breath necessary for assessing the health status of both diabetic and non-diabetic individuals. Therefore, the efficiency of different sorbent materials including Tenax, Optipore, Hayasep, and Porapak for the preconcentration of 100 ppmv of acetone was investigated (Fig. 7).

**Fig. 8:**
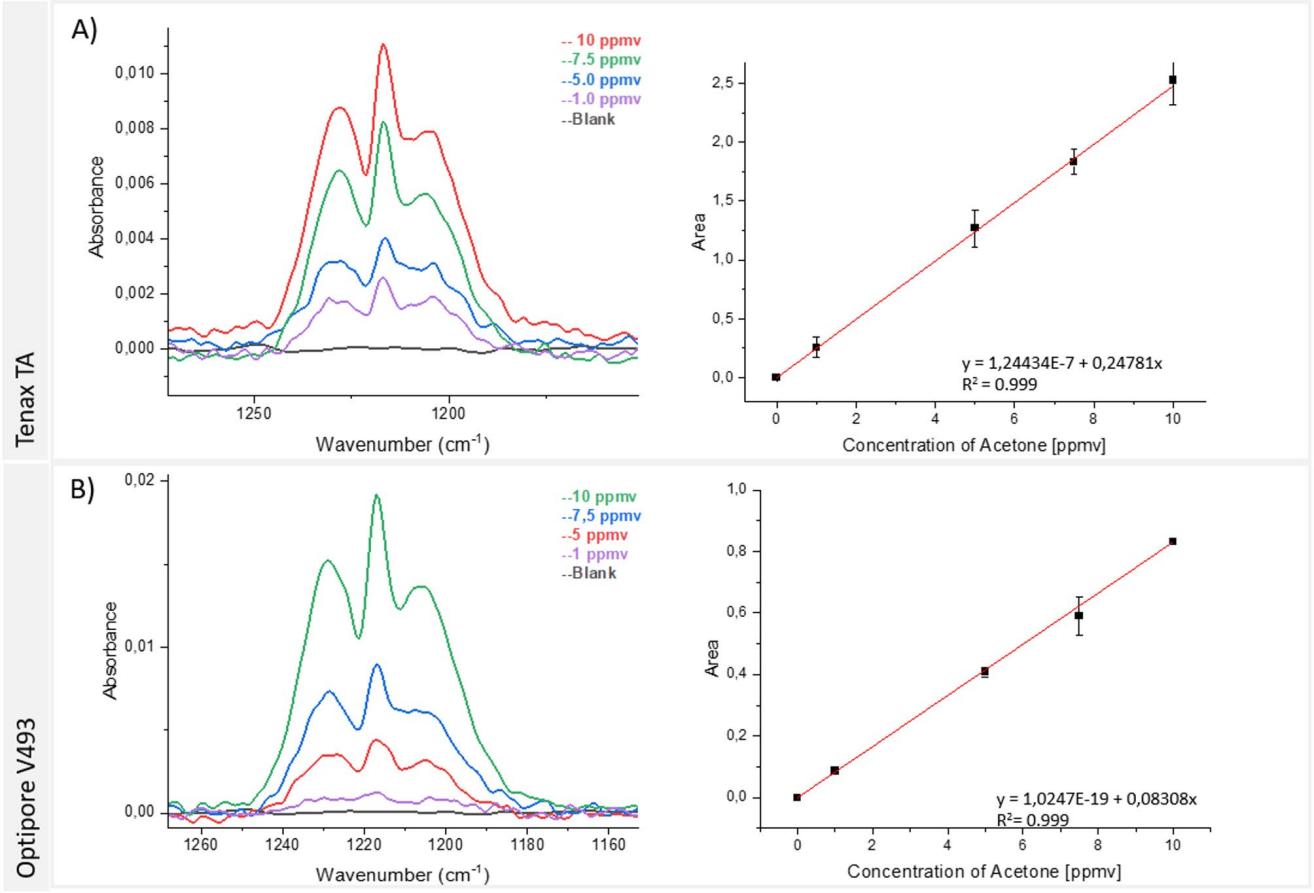
Acetone spectra with preconcentration using the muciPRECON and the MIR sensor with a 15 cm iHWG. IR spectra were obtained using (**A**) Tenax and (**B**) Optipore along with the obtained calibration functions. All the calibration curve were constructed using three measurements for each concentration point.

Porapak sorbent proved inefficient at desorbing acetone at 100 °C producing a smaller peak area compared to the results obtained without preconcentration. Given that Hayasep has a lower preconcentration factor (PCF) compared to Tenax and Optipore, the subsequent studies focused exclusively on Tenax and Optipore. The analytical performance of these absorbent materials are summarized in Fig. 8.

The analysis effectively identified Tenax as the most useful solid adsorbent at a desorption temperature of 100 °C. The PCF obtained from the ratio between the initial concentration and the concentration after the preconcentration step was > 153 (Table 2), and the material also facilitated the complete desorption of acetone at a reduced time vs. the other solid adsorbents.

**Table 2:** Performance of solid adsorbents for acetone preconcentration including PFCs, LODs and LOQs.

Sorbent	PCF	LOD (ppmv)	LOQ (ppmv)
Without preconcentration	–	15.9 ± 0.003	51.07 ± 0.004
Tenax	153.26	0.12 ± 0.0002	0.34 ± 0.0016
Porapak QS	81.53	0.19 ± 0.0014	0.63 ± 0.0008
Optipore V493	54.81	0.29 ± 0.0039	0.96 ± 0.0012
Hayesep D	51.00	0.31 ± 0.0021	1.04 ± 0.0036

### Analytical parameters

The analytical parameters of muciPRECON using Tenax TA as a sorbent material was evaluated using a calibration curve based on the peak area in the spectral region between 1260 and 1170 cm^−1^, versus acetone concentrations in the range of 1 to 10 ppmv. For each concentration level, the average of three replicate measurements was used.

The linear regression obtained from the calibration data was used to calculate the LOD and LOQ. The LOD was defined as three times the standard deviation of the blank signal divided by the slope of the calibration curve, while the LOQ was determined as ten times this relation. The precision of the method was assessed by calculating the relative standard deviation (RSD) of three independent measurements of a 5 ppmv acetone standard, which resulted in an RSD of 10%, indicating consistent repeatability under the conditions tested.

In addition, to assess the accuracy of the calibration function, a back-calculation procedure was carried out by applying the measured absorbance responses of the calibration standards to the regression model. The resulting concentrations were then compared with their corresponding nominal values. For the 1 ppmv standard, the recalculated concentration deviated by 4.0%; for 5 ppmv, the deviation was 4.96%; for 7.5 ppmv, it was 0.4%; and for 10 ppmv, 5.0%. All the relative errors were within the ± 5% range, which is well below the ± 15% limit recommended by the analytical validation guidelines^30,31^. These results confirm the suitability of the calibration model for accurate quantification throughout the concentration range tested. A summary of the analytical performance parameters is presented in Table 3.

**Table 3:** Analytical parameters of MuciPRECON for acetone quantification using preconcentration with tenax TA and mid-infrared detection.

Analytical parameter	Value
Linearity range	1–10 ppmv
LOD (ppmv)	0.12 ± 0.0002
LOQ (ppmv)	0.34 ± 0.0016
*r* ^2^	0.999
Linear equation	y = 0.2495x + 0.0053
RSD (*n* = 3)	< 10%
Accuracy	5%
Response time	6.6 min

The limits of quantification and detection for acetone using Tenax as a sorbent material are sufficient to detect the presence of acetone within exhaled breath of healthy individuals. Additionally, it is adequate to detect and quantify acetone concentrations that indicate potential physiological issues in unhealthy patients.

## Conclusions

The present study demonstrates the effectiveness of mid-infrared photonic sensor systems augmented by preconcentration strategies via solid adsorbent materials exemplified for the relevant target analyte acetone. The adsorption/desorption properties of Tenax render the material ideal to achieve preconcentration factors (> 150) pushing the limits of detection and quantification relevant for health-related application scenarios (e.g., diabetes). This suggests that factors other than surface area, such as desorption efficiency, play a crucial role in sorbent performance.

These results are promising for detecting acetone levels within the relevant physiological range commonly found in exhaled air. In addition, A promising approach to improving detectability and achieving quantification limits around 0.1 ppm is to optimize the mass of the sorbent material, providing more active sites for analyte capture, and to adjust the system parameters, such as the diameter of the preconcentration channels and the sampling time. These changes can increase capture efficiency, allowing the detection of compounds at lower concentrations. This improvement has great potential for increasing the sensitivity of the system, especially in disease diagnosis applications by analysing biomarkers in exhaled air.

Finally, it is demonstrated that compact preconcentration devices such as the muciPRECON may readily augment photonic sensing devices amplifying the analytical signals toward concentration ranges relevant in practical sensing scenarios while maintaining portability and rapid analytical response times.

## Data Availability

All datasets generated during and/or analysed during the current study are available from the corresponding author on reasonable request.
